# Novel biomarkers predict prognosis and drug-induced neuroendocrine differentiation in patients with prostate cancer

**DOI:** 10.3389/fendo.2022.1005916

**Published:** 2023-01-05

**Authors:** Jingwei Lin, Yingxin Cai, Zuomin Wang, Yuxiang Ma, Jinyou Pan, Yangzhou Liu, Zhigang Zhao

**Affiliations:** Department of Urology & Andrology, Minimally Invasive Surgery Center, Guangdong Provincial Key Laboratory of Urology, The First Affiliated Hospital of Guangzhou Medical University, Guangzhou, Guangdong, China

**Keywords:** single-cell RNA-seq, castration-resistant prostate cancer, neuroendocrine, cellular communication, prognosis

## Abstract

**Background:**

A huge focus is being placed on the development of novel signatures in the form of new combinatorial regimens to distinguish the neuroendocrine (NE) characteristics from castration resistant prostate cancer (CRPC) timely and accurately, as well as predict the disease-free survival (DFS) and progression-free survival (PFS) of prostate cancer (PCa) patients.

**Methods:**

Single cell data of 4 normal samples, 3 CRPC samples and 3 CRPC-NE samples were obtained from GEO database, and CellChatDB was used for potential intercellular communication, Secondly, using the “limma” package (v3.52.0), we obtained the differential expressed genes between CRPC and CRPC-NE both in single-cell RNA seq and bulk RNA seq samples, and discovered 12 differential genes characterized by CRPC-NE. Then, on the one hand, the diagnosis model of CRPC-NE is developed by random forest algorithm and artificial neural network (ANN) through Cbioportal database; On the other hand, using the data in Cbioportal and GEO database, the DFS and PFS prognostic model of PCa was established and verified through univariate Cox analysis, least absolute shrinkage and selection operator (Lasso) regression and multivariate Cox regression in R software. Finally, somatic mutation and immune infiltration were also discussed.

**Results:**

Our research shows that there exists specific intercellular communication in classified clusters. Secondly, a CRPC-NE diagnostic model of six genes (*HMGN2, MLLT11, SOX4, PCSK1N, RGS16* and *PTMA*) has been established and verified, the area under the ROC curve (AUC) is as high as 0.952 (95% CI: 0.882−0.994). The mutation landscape shows that these six genes are rarely mutated in the CRPC and NEPC samples. In addition, NE-DFS signature (*STMN1* and *PCSK1N*) and NE-PFS signature (*STMN1, UBE2S and HMGN2*) are good predictors of DFS and PFS in PCa patients and better than other clinical features. Lastly, the infiltration levels of plasma cells, T cells CD4 naive, Eosinophils and Monocytes were significantly different between the CRPC and NEPC groups.

**Conclusions:**

This study revealed the heterogeneity between CRPC and CRPC-NE from different perspectives, and developed a reliable diagnostic model of CRPC-NE and robust prognostic models for PCa.

## Introduction

Prostate cancer has become the second most common cancer in men worldwide, and androgen deprivation therapy (ADT) plays an indispensable impact on the treatment of PCa. On the one hand, enzalutamide, as an androgen receptor inhibitor, competes and replaces the natural ligand of androgen receptor by closely binding with the ligand binding domain of androgen receptor. At the same time, it also inhibits the translocation receptor of androgen from entering the nucleus and impairs the transcriptional activation of androgen response target genes (1). On the other hand, abiraterone weakens androgen receptor signaling by consuming adrenal and intra-tumoral androgens (2). Nevertheless, due to complex mechanisms such as lineage plasticity and phenotype switching, cytokine dysregulation (3). Prostate cancer cells can adapt to androgen deprivation and restore androgen receptor signaling, eventually progressing to CRPC, even CRPC-NE, which is a lethal subtype of PCa with extremely poor survival rate (4–6). In addition, the use of AR inhibitors is accompanied by an increase in the incidence rate of highly invasive AR negative prostate cancer. The percentage of AR negative tumors in mCRPC patients increased from 11% (1998-2011) to 36% (2012-2016) after the introduction of effective androgen receptor signaling inhibitors (such as enzalutamide and abiraterone) (7). Almost all men will eventually develop castration resistant prostate cancer (CRPC) after ADT (8), Furthermore, the most common situation is that during drug treatment, nearly 25% CRPC gradually trans-differentiate into NEPC (9), called t-NEPC, but neuroendocrine prostate cancer can also presented *de novo*.

Presently, NEPC is divided into different subtypes according to different morphological characteristics: 1. Adenocarcinoma with neuroendocrine (NE) differentiation; 2. Paneth cell NE differentiation; 3. Carcinoid; 4. Small-cell carcinoma; 5. Large-cell NE carcinoma; and 6. Mixed NE carcinoma-acinar adenocarcinoma (10). Zou et al. have shown that focal neuroendocrine differentiation (NED) and ultimately well differentiated neuroendocrine prostate cancer are directly produced by trans-differentiation of luminal adenocarcinoma cells (11), which indicates that in the process of CRPC patients treated with androgen deprivation, luminal cells inside could experience trans-differentiation, resulting in luminal/NE intermediate cells. Previous studies have shown that prostate basal cells express basal keratins *KRT5, KRT14* and key transcription factors *TP63 (* 12); Luminal or secretory cells express keratins *KRT8, KRT18*, androgen receptors, and secretory proteins consisting of prostate specific antigen (PSA) and prostatic acid phosphatase (13). An increasing number of neuroendocrine prostate cancer markers (such as *CHGB, ENO2, LMO3, EZH2, SOX2* and *SIAH2*) are being identified (14, 15). It has been reported that in mouse and adult prostate, cells with co-expression markers of basal cells and luminal cells (such as the co-expression of *KRT5/KRT14* and *KRT8/KRT18/KRT19*) are called intermediate cells, representing either pluripotent prostate stem cells or intermediate cells between basal stem cells and luminal progenitor cells (16), supplying a solid support to classify and annotate cells.

Great importance should be attached to develop diagnostic signatures for CRPC with NE characteristics. Zhang et al. has successfully identified four novel biomarkers for the diagnosis of NEPC, including *NPTX1, PCSK1, ASXL3*, and *TRIM9* (17) *via* Bulk-RNA sequencing data, in our study, by combining single-cell RNA seq with Bulk-RNA seq, the CRPC-NE diagnostic model *via* machine learning algorithm was successfully built, and the prostate cancer prognosis model was also constructed and validated triumphantly.

## Materials and methods

### Data collection and procession of Sc-RNA seq and Bulk-RNA seq

Attaching great attention on neuroendocrine prostate cancer, the sample inclusion criteria are as follows: (1) the patients must have developed resistance to castration therapy; (2) Gene expression data must be available for both CRPC and NEPC tumors; (3) The diagnostic information must be clear. The single-cell RNA sequencing information of GSE176031 (18) as well as GSE137829 (19) were obtained *via* GEO database(https://www.ncbi.nlm.nih.gov/geo/), The former provides with 4 normal samples (8038 cells) taken from radical prostatectomies, The single-cell transcriptome information of NEPC and CRPC were obtained from the other one, including 3 CRPC samples (7119 cells) and 3 NEPC samples (16384 cells). Harmony algorithm was not used to remove batch effects so as not to eliminate the inherent differences between samples. Then CRPC and CRPC-NE clusters were separated according to well-acknowledged cell markers, We used CellChat (v1.4.0) R package to analyze the intercellular communication among annotated clusters (20), and calculated 102 differentially expressed genes (DEGs) (logFC > 0.5 & pvalue < 0.05) between CRPC and CRPC-NE by “FindMarkers” function in Seurat (v4.1.1) R package (21–24). These genes were then used for GO and KEGG analysis.

**Figure 1:**
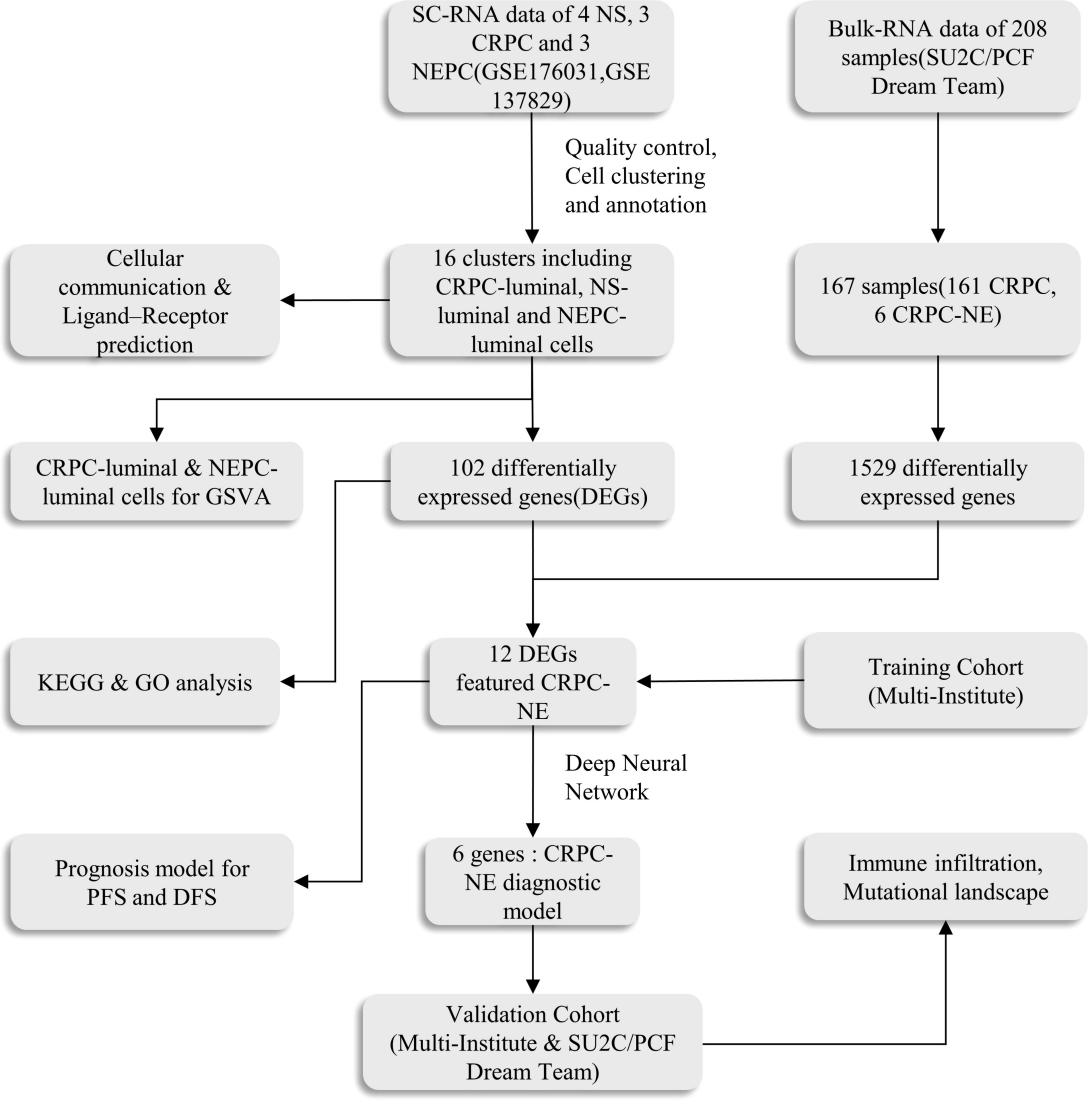
The main workflow of the study. SC-RNA, single-cell RNA; NS, normal sample; CRPC, castration-resistant prostate cancer; NEPC, Neuroendocrine prostate cancer;DEGs, differentially expressed genes; GSVA, Gene Set Variation Analysis; GO, Gene Ontology; KEGG, Kyoto Encyclopedia of Genes and Genomes; ROC, receiver operating characteristic; DFS, disease free survival; PFS, progression free survival.

**Figure 2:**
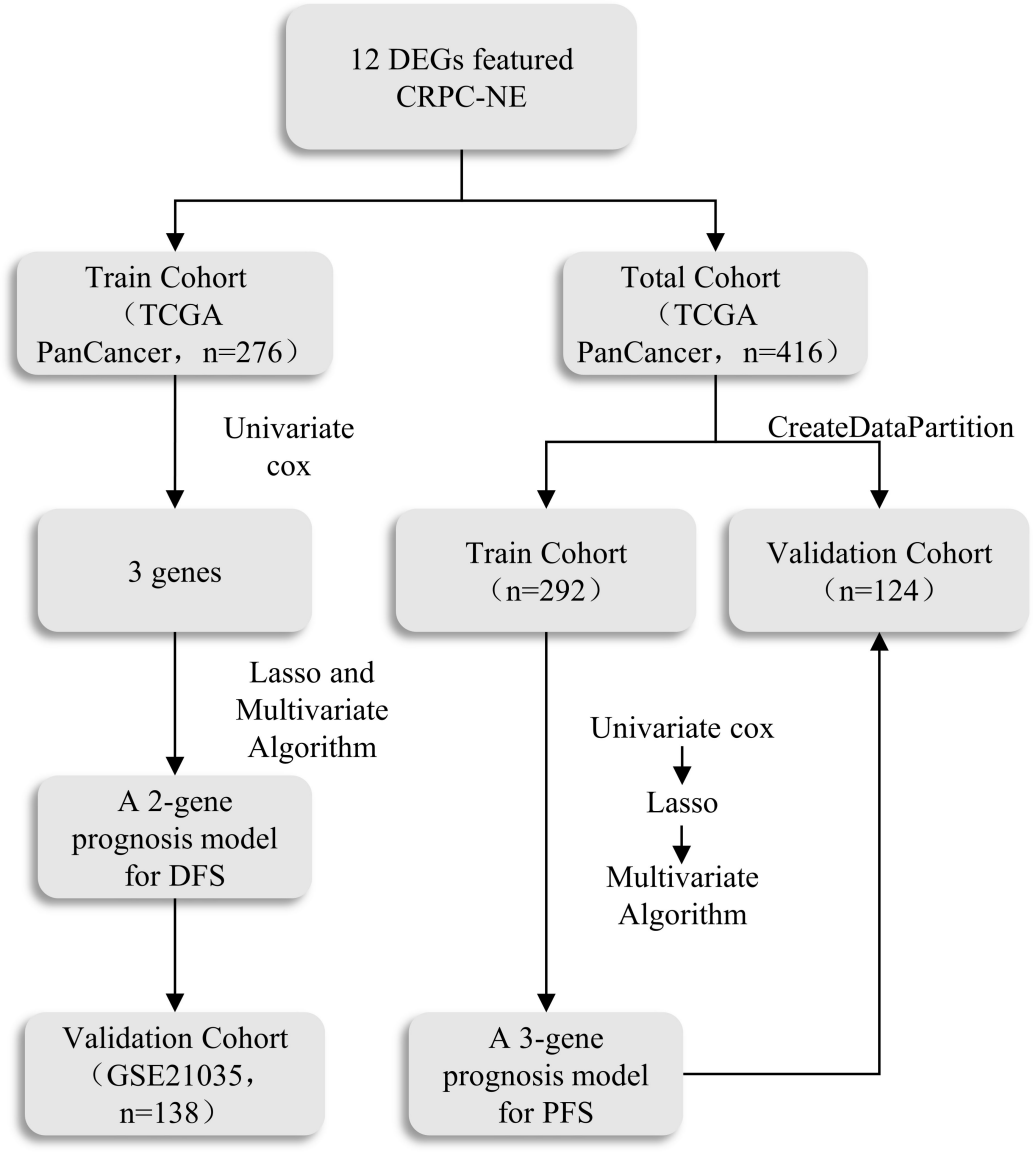
The scheme of the prognosis model.

The Bulk transcriptome RNA-seq data and corresponding clinical data, consisting of SU2C/PCF Dream Team(n=208) (25), Multi-Institute Cohort (n=49) (26) were download from Cbioportal Database (https://www.cbioportal.org/) and used to identify genes upregulated in CRPC-NE samples compared with CRPC samples after quality control, 41 samples were excluded due to inadequate information in SU2C/PCF Dream Team cohort. Only 12 genes highly expressed in both single-cell transcriptome data and Bulk-RNA data were selected for the establishment of CRPC-NE diagnosis model. The workflow of the diagnostic model is presented in **Figure 1**. Additionally, TCGA PanCancer data (27) from Cbioportal Database and 138 PCa samples in GSE21035 (28) were explored in order to construct prognosis model for DFS as well as PFS. The workflow of the prognosis model is demonstrated in **Figure 2**. Genes mapped to multiple probes were calculated by their average values. The batch effects of Bulk RNA-seq data were modified through “ComBat” function in sva (v3.44.0) package (29). The clinicopathological information of enrolled samples is listed in **Table 1**.

**Table 1:** Characteristics of sample cohorts used for the analysis of DFS as well as PFS.

Characteristics		DFS cohort		PFS cohort	
		TCGA (n=276)	GSE21035 (n=138)	TCGA-train (n=292)	TCGA-validation (n=124)
Age (year)					
≤65		121	115	207	85
>65		155	23	85	39
PSA (ng/ml)					
≤10		NA	112	NA	NA
>10		NA	24	NA	NA
Not available		NA	2	NA	NA
Gleason score					
≤6		NA	77	NA	NA
7		NA	48	NA	NA
≥8		NA	13	NA	NA
Disease-free event		248	103	NA	NA
Progression event		NA	NA	63	21
T-stage					
T1/T2		121	86	104	38
T3/T4		155	52	188	86
N-stage					
N0		246	103	239	99
N1		30	12	53	25
Nx		NA	23	NA	NA
Surgery-type					
RP		NA	98	NA	NA
Others		NA	40	NA	NA
Radiation therapy					
Yes		32	18	12	18
No		242	NA	231	92
Not available		2	120	35	14

### Single−cell RNA−seq analysis

The Seurat package (v 4.1.1) was utilized to generate the object and filtered out cells with poor quality. Then, we conducted standard data preprocessing, where we calculated the percentage of the gene numbers, cell counts and mitochondria sequencing count. Genes with less than only 3 cells detected and disregarded cells with less than 50 detected gene numbers were excluded. We filtered out cells with fewer than 500 or more than 4,000 detected genes and those with a high mitochondrial content (>5%). After discarding poor-quality cells, a total of 12,165 cells were retained for downstream analysis. To normalize the library size effect in each cell, we scaled UMI counts using scale.factor = 10,000. Following log transformation of the data, other factors, including “percent.mt”, “nCount_RNA” and “nFeature_RNA”, were corrected for variation regression using the “ScaleData” function in Seurat (v 4.1.1). The corrected-normalized data metrics were applied to the standard analysis as described in the Seurat R package. The top 1,500 variable genes were extracted for principal component analysis (PCA). The top 30 principal components were kept for Uniform Manifold Approximation and Projection for Dimension Reduction (UMAP) visualization and clustering. We performed cell clustering using the “FindClusters” function (resolution = 0.3) implemented in the Seurat R package. Afterwards, the clusters were verified by SingleR package (v1.10.0) and canonical markers (30). Moreover, we utilized”FindAllMarkers” function to identify marker genes between cluster “CRPC_Luminal” and “NEPC_Luminal/NE” with the filter value of absolute log2 fold change (FC) ≥ 0.5 and the minimum cell population fraction in either of the two populations was 0.25 (31).

### Pseudotime trajectory analysis

Importantly, after passing quality control, Pseudotime and trajectory analysis of single cells were performed *via* “monocle” R package (v2.24.0) (32–34), genes were placed into the Reversed Graph Embedding algorithm of Monocle to shape the trajectory. Then, Monocle applied a dimensionality reduction to the data and ordered the cells in pseudotime.

### Ligand–receptor expression and cell interactions

Cell-to-cell communication “CellChat” (v1.4.0) R package was ascertained by evaluating expression of pairs of ligands and receptors within cell populations, thus to reveal the potential interaction between various cells types. Gene expression of 0.2 was set as the valid cutoff point. The specific signaling pathways were selected for further visualization so as to reveal the strength of specific pathways among 16 clusters. In addition, the potential ligand-receptor interaction between luminal/NE cells and other cells was also explored.

### Functional analyses and mechanism exploration

Firstly, Gene Set Variation Analysis (GSVA) was performed with the GSVA package (v1.44.0) of R software with default parameters (35). The list of KEGG terms was obtained from the Gene Set Enrichment Analysis database (https://www.gsea-msigdb.org/gsea/msigdb/genesets.jsp?collection=CP : KEGG).

Furthermore, the DEGs between CRPC-luminal & NEPC-luminal clusters were identified with R package limma (v3.52.0) (36). Then the pathway enrichment analyses, including Gene Ontology (GO) analysis and KEGG analyses were completed to explore distinct pathways (37–39).

### Random forest algorithm and artificial neural network model for diagnosis model

A random forest algorithm was applied on 49 samples (Multi-Institute Cohort) from Cbioportal to find the most important genes associated with the phenotype. Briefly, We utilized randomForest R package (v4.7-1.1) to find the most important genes associated with diagnosis status in CRPC and CRPC-NE samples (40). The genes whose “MeanDecreaseGini” > 1 were choose to build the artificial neural network (ANN) model. Based on multilayer perceptron network (MLP), the ANN model consists of input nodes, hidden layers, and an output node (41), In our study, six genes (*HMGN2, MLLT11, SOX4, PCSK1N, RGS16* and *PTMA*) were selected as the input nodes, and one indicator (with or without neuroendocrine differentiation) was used as the output node (42). Consequently, the diagnosis model was validated in samples from Multi-Institute and SU2C/PCF Dream Team (n=216) downloaded from Cbioportal. The sensitivity and specificity of the diagnostic models were evaluated by the receiver operating characteristic (ROC) curves (43).

### Construction and validation of prognostic model for DFS and PFS

By comparing CRPC with CRPC-NE *via* “limma” (v 3.52.0) R pacakge, 12 genes highly expressed in both single-cell transcriptome data and Bulk-RNA data were discovered. To begin with, SU2C/PCF Dream Team (n=276) in the Cbioportal dataset were regarded as training cohort. NEPC characteristic genes were analyzed by univariate Cox to obtain candidate prognostic genes (P<0.05), Subsequently, the least absolute shrinkage and selection operator (LASSO) method by “glmnet” (v4.1-4) R package was used to minimize overfitting risk (44), and select the optimal gene combination with the lowest Akaike information criteria (AIC) in a Stepwise Algorithm, Finally, a 2-gene prognostic signature (NE-DFS signature) for DFS was built based on the regression coefficient derived from the multivariate Cox regression model and the optimized genes. The formula are as follows:


$$NE-DFS\;signature\;score=\sum_{i=1}^{n}\left(\beta_{i}*\exp i\right)$$

where n was the number of enrolled genes, βi represented the coefficient of the gene and Exp *i* was the candidate gene’s expression level. Then, patients were classified into high- and low- risk groups according to the median, the Kaplan–Meier plot and log-rank test were applied to evaluate differences between the high-risk and low-risk subgroups by the R package “survival” (v3.3-1) (45). The receiver operating characteristic (ROC) curve performed by “timeROC” (v 0.4) R package was used to judge the efficiency of the NE-DFS signature,

Afterwards, we validated the model in the GSE21035 (n=138) cohorts. Data from different platform were modified through “ComBat” function in sva (v3.44.0) package to eliminate batch effects. Similarly, A 3-gene prognosis model for PFS was constructed and validated in TCGA PanCancer cohort (n=416). 416 PCa patients in the dataset were randomly assigned to training (n = 292) and internal validation cohort (n = 124) at a 7:3 ratio, the remaining has been described in detail above.

### Immune infiltration and tumor mutational burden exploration

Normalized expression levels (Affymetrix intensity) of gene signatures that distinguish 22 immune cell types from each other and other cell types was downloaded from the Supplementary Table 1 of this article (46), namely LM22 signature. Then we identify the proportions of the 22 immune cells from each sample by “CIBERSORT”. The algorithm was run using the LM22 signature and 1000 permutations. For each sample, the final CIBERSORT output estimates were normalized to sum up to one. The Wilcoxon rank-sum test was used to compare the expression differences of 22 types of immune cells between CRPC and CRPC-NE patients. Only cases with a CIBERSORT output of p < 0.05 were considered to be eligible for subsequent analysis and visualization. Additionally, waterfall plots were generated to explore the mutation characteristics of the 12 CRPC-NE featured markers by “maftools” (v2.12.0) package (47).

### Nomogram construction

Nomogram analysis was constructed in the training group to predict the outcome of the individual. The upper part is the scoring system and the lower part is the prediction system. The 1-, 2-, 3- and 5-year survival rate of PCa patients could exactly be predicted by total points of every factor. Verification of the prediction accuracy of DFS and PFS was performed in patients of the validation group.

### Statistical analyses

Besides the Venn diagrams were drawn online (https://bioinformatics.psb.ugent.be/webtools/Venn/). The other statistical analyses and visualization were conducted using the R software (v4.2.0) and Bioconductor (v3.15). Statistical differences between the two groups were assessed using the Wilcoxon test. P < 0.05 was considered statistically significant.

## Results

### Single−cell RNA−seq profiling, clustering and markers

**Figure 3:**
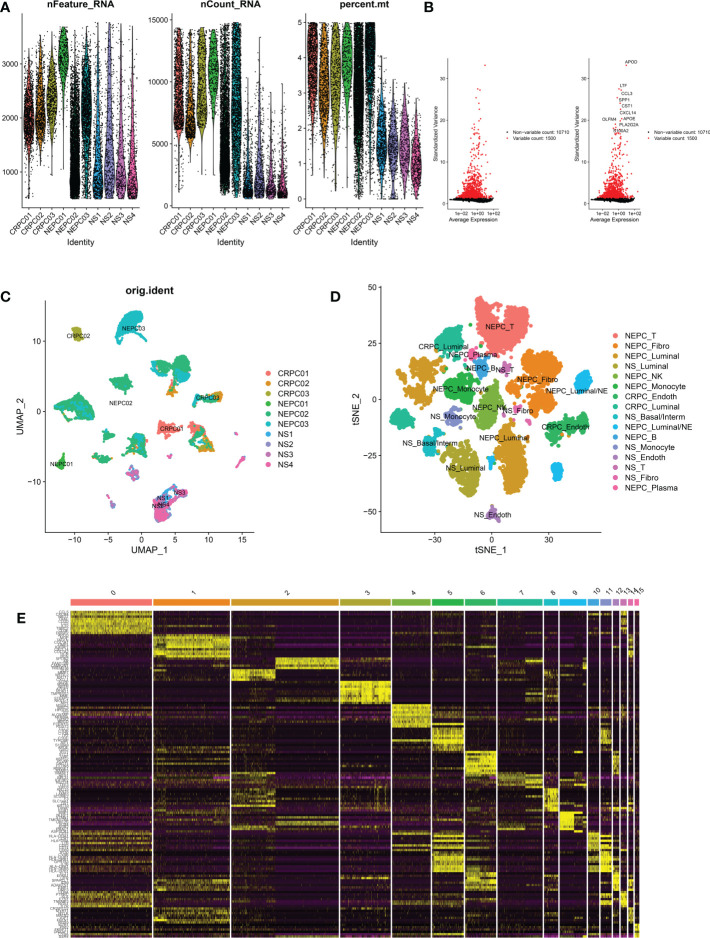
Analysis of single-cell RNA seq of 3 CRPC tissues, 3 NEPC tissues and 4 NS tissues. CRPC, castration resistant prostate cancer; NEPC, neuroendocrine prostate cancer; NS, negative samples; Fibro, Fibroblast; Basal/interm, Basal/intermediate; Endoth, endothelial; **(A)**: the number of RNA features (nFeature_RNA) and absolute UMI counts (nCount_RNA) after quality control filtering of each cell. **(B)**: We explored 1,500 high variable genes that exhibit high cell-to-cell variation, and the names of the top 10 genes are marked. **(C)**: Using UMAP dimensionality reduction algorithm, 12165 cells from 10 samples were displayed. **(D)**: Cells were classified into 16 clusters *via* t-SNE dimensionality reduction algorithm based on the source of the cluster, each cluster was marked with the source of the cluster plus the annotated cell types. There may exist 2 same cell types in 16 clusters. **(E)**: Heatmap depicting expressions of top 10 marker genes among 16 clusters.

Two Sc-RNA seq datasets (GSE176031 and GSE137829) in the GEO database were used to obtain normal samples (8038 cells), CRPC samples (7119 cells) and NEPC samples (16384 cells). After initial quality control assessment, 12,165 high-quality cell samples isolated from three distinguished types of tissues were screened and illustrated for further analyses (**Figure 3A**). 1,500 high variable genes and the names of the top 10 genes are marked in **Figure 3B**. Principal component analysis (PCA) and UMAP was used for preliminary dimension reduction of Sc-RNA seq data (**Figure 3C**). We subsequently apply t-distributed stochastic neighbor embedding (t-SNE) algorithm on the top 30 principal components to visualize the high dimensional scRNA-seq data, and successfully classified cells into 10 clusters (T cell, Fibroblast, Luminal, NK cell, Monocyte, Endothelial, Basal/Interm, Luminal/NE, B cell, Plasma) by previous canonical cell marker combined with “SingleR” package (v1.10.0), which were later annotated to acknowledged 16 cell types (**Figure 3D**) according to the sample (**Table 2**). It can be seen that not all luminal cells in 3 NEPC samples have the characteristics of neuroendocrine differentiation. The cluster “NEPC_Luminal/NE” has neuroendocrine features, while cluster “NEPC_Luminal” does not. **Figure 3E** illustrates the heatmap of marker gene expression in 16 clusters.

**Table 2:** Cell cluster distribution and cell marker.

Cell Cluster	Cell marker	Cell Type
0, 13	CD3D, IL7R, TRBC2, CCL5, CCL4, CD8A, CXCR4, ETS1, CD69	T cell
1, 14	DCN, LUM, PTN, APOD, IGFBP5, CCDC80, CFD, LTBP4, COL1A2, FBLN1, MEG3	Fibroblast
2, 3, 7	KRT19, KRT8, KRT18, AR	Luminal
4	NKG7, GNLY, KLRD1, KLRB1, FGFBP2, PRF1, CD8A, CD8B, GZMH, GZMA	NK cell
5, 11	S100A9, EREG, NEAT1, TKT, THBS1, TSPO, CSTA	Monocyte
6, 12	TM4SF1, RNASE1, EGFL7, RAMP3, PLVAP, ECSCR, FKBP1A, EMP1, VWF, EMCN	Endothelial
8	KRT5, KRT19, KRT8, KRT18	Basal/Interm
9	CHGB, ENO2, LMO3, EZH2, SOX2, SIAH2	Luminal/NE
10	CD22, CD79B, LY9, CCR7, IRF8, CD83, BTG1, BANK1	B cell
15	SEC11C, XBP1, PRDX4, SPCS2, SSR3, SDF2L1, MANF, TMEM258, DNAJB9	Plasma

**Figure 4:**
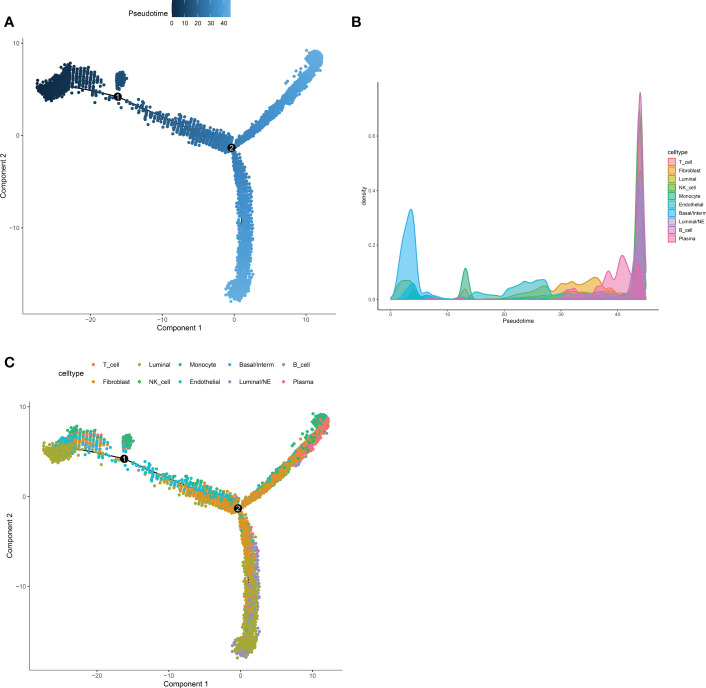
Pseudotime and trajectory analysis revealed the tendency curve among various cell types. **(A)** The pseudo time is shown as the depth of the color, the darker the blue, the smaller the pseudo time, which means that the cells appear earlier. The dots above represent cells. **(B, C)** Pseudotime-Density diagram demonstrated cells including immune cells (such as NK-T cell, B cell, Plasma) as well as Luminal/NE cells gather around the destination. X-axis means the value of principal component 1 (the first principal direction of maximum sample change) and Y-axis means the value of principal component 2.

Next, Pseudotime and trajectory analysis were conducted *via* “monocle” package (v 2.24.0) to explore the potential cellular evolution. The predicted pseudotime trajectory began from the upper left and stretched as cells approach the up and bottom right branches (**Figure 4A**). Intriguingly, cells including fibroblast, luminal, basal/interm as well as Endothelial were mainly localized in the early stages of pseudotime trajectory while immune cells (NK-T cell, B cell, Plasma) with Luminal/NE cells moved towards the termini, implying that T and B cells, as momentous components of tumor microenvironment, may play an indispensable role in the occurrence and development of CRPC and NEPC (**Figures 4B, C**).

### Identification of CRPC-NE featured markers

**Figure 5:**
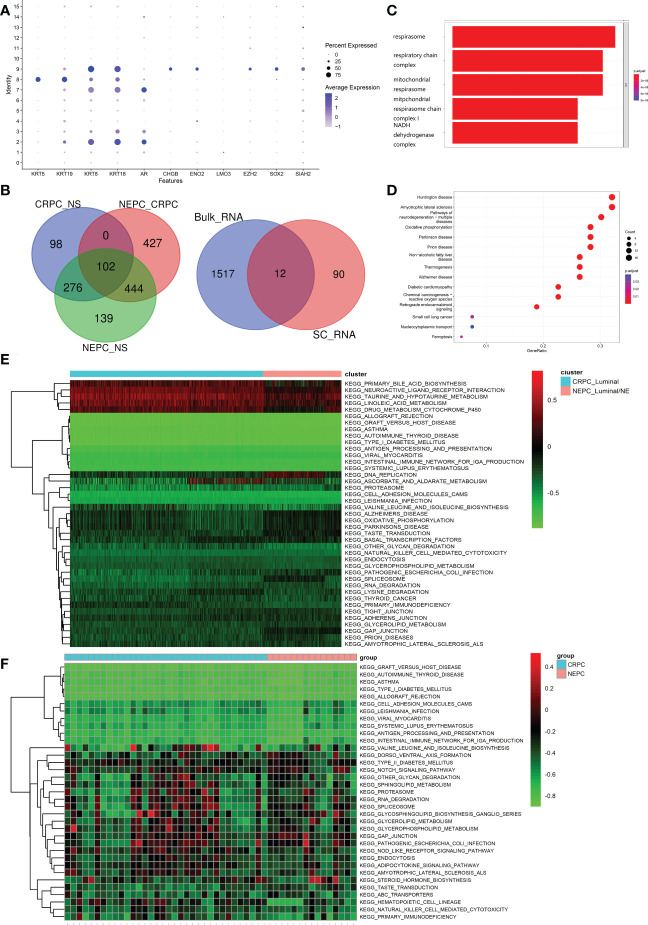
Identification and functional analysis of CRPC-NE featured markers. **(A)** Marker gene expression for epithelial cells (KRT5, KRT19, KRT8, KRT18, AR) and neuroendocrine characteristic cells (CHGB, ENO2, LMO3, EZH2, SOX2, SIAH2), in which dot size and color represent percentage of marker gene expression and the averaged scaled expression value, respectively. **(B)** 102 genes with higher expression in NEPC than that in CRPC, and the latter is higher than that in NS were screened out. Then we selected genes shared between the SC-RNA data (102 genes) and Bulk-RNA data (1529 genes). **(C, D)** GO enrichment and KEGG pathway enrichment analysis of differentially expressed 102 genes. **(E, F)** Heatmap illustrating the differential KEGG pathways (upper panel) between CRPC_Luminal cluster and NEPC_Luminal/NE cluster at the single cell RNA-seq level, and discrepant KEGG pathway (lower panel) from the aspect of Bulk-RNA seq. The color indicates the level of pathway expression.

As set forth in the article, 16 clusters were identified, **Figure 5A** exhibits the specific markers of basal, Luminal and NE of PCa. A total of 102 genes were identified as DEGs (LogFC>0.5 & pvalue<0.05), which were higher regulated in NEPC_Luminal/NE cluster, namely NEPC cells, than that in CRPC_Luminal and NS_Luminal cluster. Analogously, A Bulk-RNA data consisting of 167 samples (161 CRPC, 6 CRPC-NE) produces 1,529 DEGs (LogFC>0.25 & pvalue<0.05) *via* R package limma (v3.52.0). We selected genes shared between the 102 and 1529 genes (**Figure 5B**). GO analysis revealed that the 102 DEGs were mainly enriched in the biological processes of the biological oxidation process in mitochondria (**Figure 5C**). KEGG analysis indicated that the DEGs were mainly enriched in a variety of neurological diseases including Huntington disease, Amyotrophic lateral sclerosis, Pathways of neurodegeneration−multiple diseases and Oxidative phosphorylation (**Figure 5D**). To further investigate the potential pathway differences between NEPC and CRPC, and thus explain the causes of phenotypic differences between them. GSVA on the scRNA-seq data was conducted (**Figure 5E**). In contrast with CRPC-luminal, five pathways (KEGG_NEUROACTIVE_LIGAND_RECEPTOR_INTERACTION, KEGG_PRIMARY_BILE_ACID_BIOSYNTHESIS, KEGG_TAURINE_AND_HYPOTAURINE_METABOLISM, KEGG_LINOLEIC ACID METABOLISM, KEGG_drug_metablism_cytochrome_p450) were obviously down-regulated in NEPC-luminal cells. Nevertheless, distinctively differential KEGG pathways except the above fives were observed in the bulk-RNA data Multi-Institute cohort, which contains 34 CRPC and 15 CRPC-NE samples (**Figure 5F**).

### The exact ligand–receptors among different cell types

**Figure 6:**
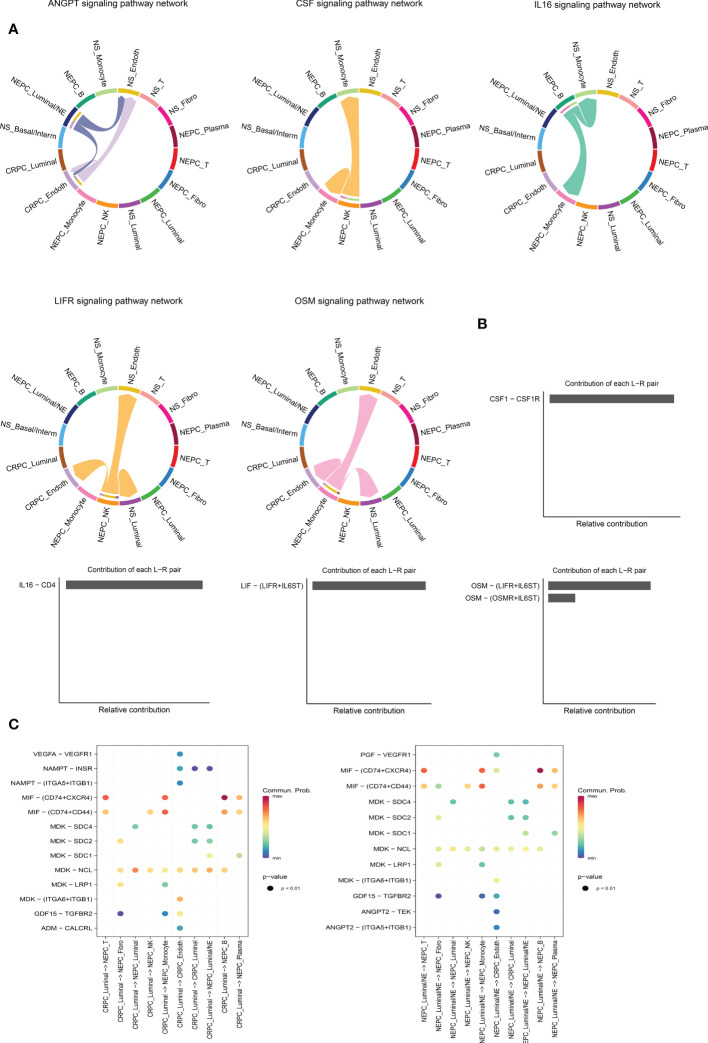
Intercellular ligand–receptor prediction among differernt clusters. **(A)** The chord diagram shows the expression of ANGTP, IL16, CSF, LIFR and OSM pathways among different cell clusters. In the peripheral ring, different colors represent different cells, Cells that send the arrow express the ligand, and cells that the arrow points to express the receptor, the more ligand-receptor pairs, the thicker the line. **(B)** Relative contribution of each ligand-receptor pair to the signal pathway, which may affect the overall communication network of the signaling pathway. CSF, IL16, LIFR and OSM pathways are shown in turn. **(C)** The extensive ligand-receptor mediated cellular interaction between different cell clusters of CRPC and NEPC has been further explored and demonstrated. The color gradient indicates the probability of cellular communication.

It is worthy of exploring the ligand–receptors interactions among 16 clusters, especially the interactions between CRPC and NEPC, we applied CellChat to infer and analyze intercellular communication networks. CellChat revealed a number of crucial ligand–receptor pairs and signaling pathways, including ANGTP, IL16, CSF, LIFR and OSM pathways (**Figure 6A**), displaying the Luminal/NE cluster regulate CRPC_Endoth and NS_ Endoth clusters through ANGTP signaling pathway, while NS_Fibro cluster displayed vast communication with other cells such as NS_Monocyte, NS_Basal/Interm, CRPC_Endoth, NS_Luminal and NEPC_Luminal clusters (mainly those featured with epithelial and endothelial markers). Intriguingly, NEPC_B cluster and NEPC_NK cluster regulate Monocyte cluster through pathways CSF and IL16, respectively, hinting the role of immune intercellular crosstalk is vital. Similarly, cluster NEPC_NK is extensively associated with endothelial and epithelial cells *via* pathways LIFR and OSM. The contribution of each ligand-receptor was showed in (**Figure 6B**), Notably, the most significant L-R pairs of CSF pathway was CSF1 − CSF1R, previous study has revealed that the CSF1/CSF1R signaling axis has been implicated in prostate cancer oncogenesis and CSF1R blockade lowered (tumor associated macrophage) TAM-induced tumorigenic factors and delayed the emergence of CRPC (48). Besides, tumor-associated macrophage accelerates the survival of CRPC cells upon docetaxel chemotherapy *via* the CSF1/CSF1R-CXCL12/CXCR4 axis (49). We further investigated the specific ligand–receptor interactions among different cell clusters, Particular attention was paid to the interactions of CRPC_Luminal and NEPC_Luminal/NE clusters with other cluster cells (**Figure 6C**). Distinct cell interactions among luminal/NE, luminal cells as well as other clusters were detected, consisting of MIF − (CD74+CXCR4), MDK − NCL and MDK − LRP1, which might participate in the formation of CRPC or NEPC through relevant channels.

### Six−gene diagnostic NEPC signature construction and verification

**Figure 7:**
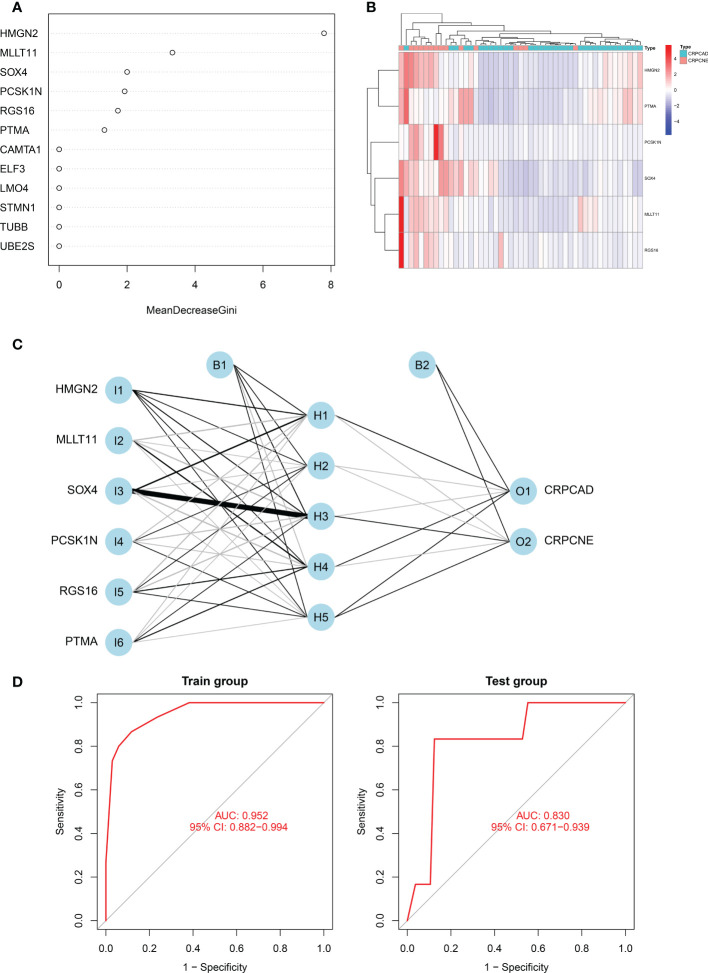
Identification of the markers to establish CRPC-NE diagnostic model. **(A)** The figure shows the weight of 12 genes to elucidate the importance of genes to disease classification (CRPC-NE or CRPC). The larger the”MeanDecreaseGini”index, the more likely this gene is to be classified as a characteristic gene. **(B)** Heatmap visualizing the expression levels of the six CRPC-NE diagnostic genes in the Cbioportal training cohort. **(C)** Results of neural network visualization: six CRPC-NE diagnostic genes were selected as the input nodes. Positive weights are connected by black lines, negative weights are connected by gray lines, and the thickness of the lines reflects the weight value. **(D)** The receiver operating characteristic (ROC) curves of 6-gene CRPC-NE diagnostic model in training cohort and validation cohort.

Firstly, in the training cohort (n=49), we applied the randomForest algorithm to analyze 12 NEPC-featured genes, the number of trees was set as 500 based on the relationship plot between the model error and the number of decision trees, and obtained the most 6 significant genes associated with the phenotype according to the value of “MeanDecreaseGini” (**Figure 7A**), which reflects the importance of genes. Then k-means unsupervised clustering was utilized to cluster the training cohort with these 6 critical factors (*HMGN2, MLLT11, SOX4, PCSK1N, RGS16* and *PTMA*) (**Figure 7B**).

In this study, The Multi-Institute cohort was used to build an artificial neural network model using the neural net package. The maximum and lowest data values were normalized before the computation began, and the number of hidden layers was set to 5, the above six genes were selected as the input nodes, and one indicator (with or without neuroendocrine differentiation) was used as the output node **Figure 7C**. The validation set was utilized to test the model score’s classification performance using the expression of genes and gene weight. So far, the diagnosis model was validated in samples from Multi-Institute and SU2C/PCF Dream Team datasets. The sensitivity and specificity of the diagnostic models were evaluated by the receiver operating characteristic (ROC) curves, nearly 0.952 (95% CI: 0.882−0.994) in the train group, indicating that it was robust. The area under the ROC curve (AUC) remains 0.830 (95% CI: 0.692−0.964) in the dataset of SU2C/PCF Dream Team from Cbioportal (**Figure 7D**).

### Immune infiltration and tumor mutational burden analysis

**Figure 8:**
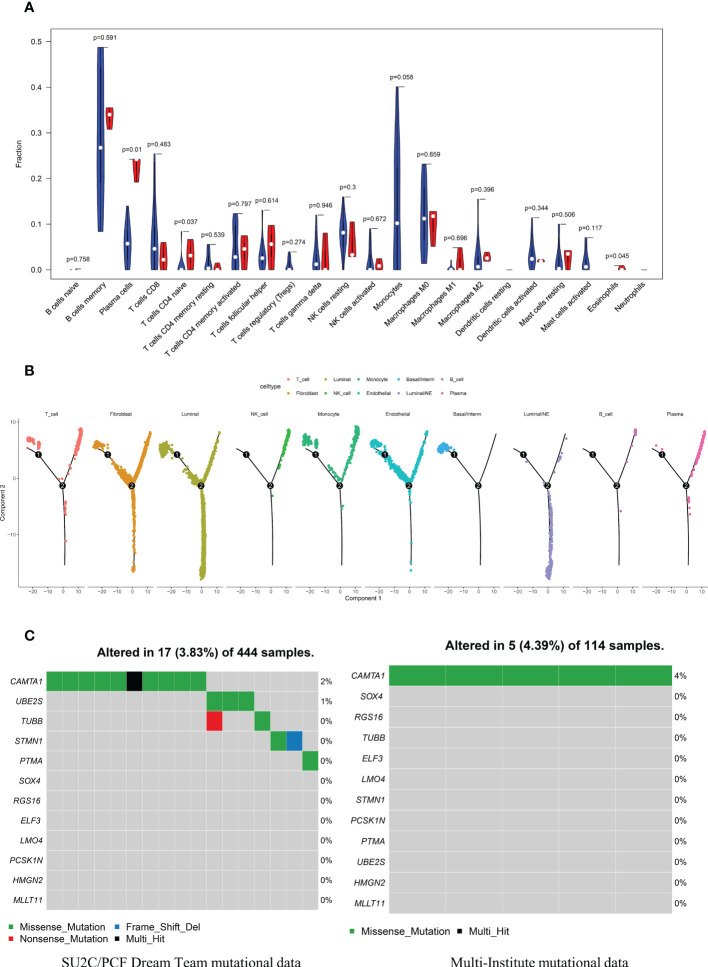
The distribution landscape of immune cell, and TMB pattern between CRPC and CRPC-NE **(A)** The difference of 22 immune infiltration between CRPC and CRPC-NE groups, red color indicates the abundance of immune cells in the latter, blue color indicates the abundance in the former. **(B)** Pseudotime trajectory analysis elucidated luminal/NE cluster and immune cells like NK, T, B and Plasma cells moved towards the termini of the trajectory. **(C)** Waterfall plots summarize the mutation landscape of 12 CRPC-NE featured genes in CRPC and CRPC-NE samples, showing that the mutation rate of these genes is low except CAMTA1.

CIBERSORT algorithm was adopted to estimate the abundances of member cell types in a mixed cell population, using gene expression data including 34 CRPC samples and 15 NEPC samples from Multi-Institute cohort (n=49). We used Wilcoxon rank-sum test to explore whether there was a difference in the expression of immune cells between the two groups, The results demonstrated that the infiltration levels of plasma cells, T cells CD4 naive, Eosinophils and Monocytes were significantly different in the two groups (**Figure 8A**). Particularly, the infiltration levels of plasma cells, T cells CD4 naive, and Eosinophils were significantly higher in cluster CRPC-NE. On the contrary, cluster CRPC appeared higher infiltration levels of Monocytes cells. Combined with the Pseudotime and trajectory of immune cells (**Figure 8B**), we could conclude that CRPC-NE is closely related to T and plasma cells in the tumor microenvironment, providing a new direction for CRPC-NE immunotherapy. Furthermore, waterfall plot revealed except for genes *CAMTA1*, few mutations were observed of the other 11 CRPC-NE featured genes in CRPC and CRPC-NE samples (**Figure 8C**).

### The prognostic model for DFS and PFS

**Figure 9:**
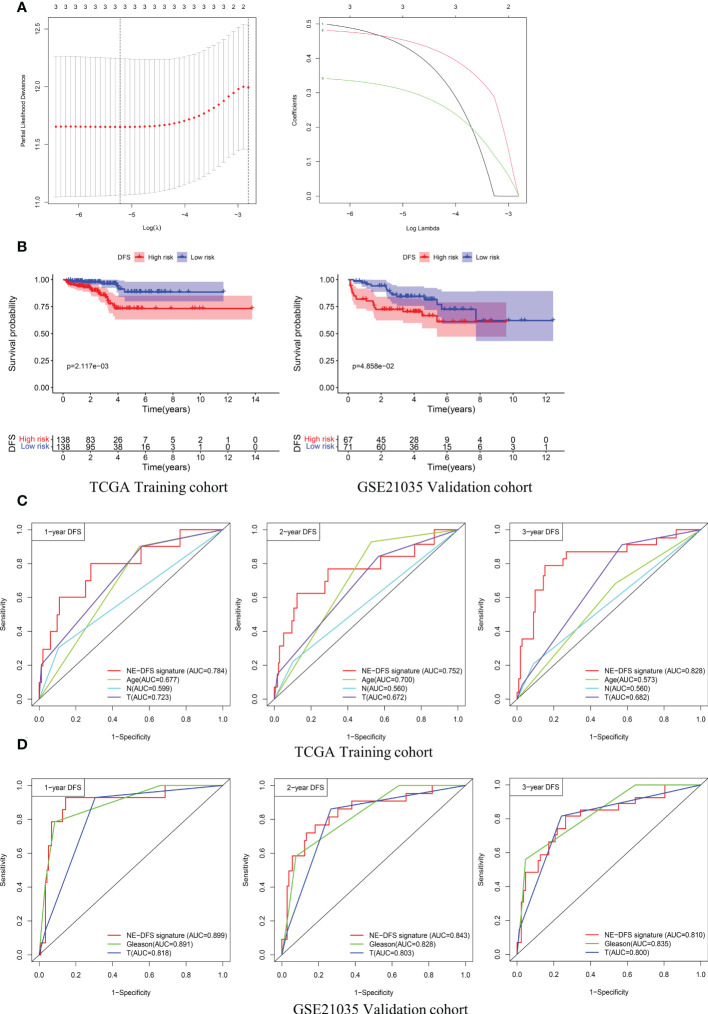
A 2-gene prognosis model for DFS (NE-DFS signature) in the TCGA PanCancer training cohort and GSE21035 validation cohort in PCa. **(A)** Three genes significantly correlated with DFS were identified through LASSO regression analysis and ten-fold cross-validations for screening of the optimal parameter lambda **(B)** Kaplan–Meier curves displayed that high-risk group exhibited worse DFS than low risk group in TCGA PanCancer training group (n=276) and GSE21035 group (n=138). **(C, D)** Receiver operating characteristic (ROC) curves of the NE-DFS signature had better Predictive effectiveness than age, tumor stage and lymph node status to evaluate the predictability of DFS at 1-, 2- and 3- year in the TCGA PanCancer training cohort, similar phenomena were observed in the GSE21035 validation group.

Univariate analysis was performed to assess associations between 12 DEGs featured CRPC-NE and DFS in the TCGA PanCancer dataset (n=276). According to the selection criteria, 3 DFS associated genes with P<0.05 were screened out for LASSO Cox regression algorithm to ensure the robustness of the prognostic model, afterwards, the lambda.min was determined as the optimal lambda value by tenfold cross-validations, the above 3 prognostic genes with non-zero coefficients were all enrolled (**Figure 9A**). subsequently, multivariate analysis and Stepwise Algorithm were used to ensure that Akaike information criterion (AIC) is the minimum, thus generating the appropriate gene combination of 2 genes (*STMN1* and *PCSK1N*) with P<0.05, namely NE-DFS signature. On the basis of the coefficients, the risk score was confirmed: NE-DFS signature score = expression level of 0.696 * *STMN1* + expression level of 0.432* *PCSK1N*. According to the median cutoff value of the score, patients were separated into high- and low-risk groups. Kaplan-Meier plots elucidated that the patients with lower scores had better DFS (**Figure 9B**), p < 0.05). Then the potential accuracy of the model was further assessed by the “timeROC” package in the training cohort, with 1-, 2- and 3-year AUCs of 0.784 (95% CI: 0.631−0.938), 0.752 (95% CI: 0.588−0.916) and 0.828 (95% CI: 0.722−0.935) respectively, better than those of Gleason scores and pathological tumor stages (**Figure 9C**).

External dataset GSE21035 (n=138) were enrolled as validation cohort to evaluate the robustness of the training group. Similarly, the samples were classified into high risk and low risk groups based on median risk score. Kaplan-Meier survival plots revealed that there is a significant difference between the high risk and low risk (p<0.05) (**Figure 9B**). The AUCs of 1-, 2- and 3- year were 0.899 (95% CI: 0.806−0.992), 0.843 (95% CI: 0.746−0.941) and 0.810 (95% CI: 0.712−0.907) respectively (**Figure 9D**), demonstrating fabulous predictive potential especially for the DFS within 3 years.

Furthermore, analogous methods were utilized to construct a 3-gene prognostic model for PFS by using TCGA PanCancer (n=416). The Total Cohort were randomly assigned to training (n = 292) and internal validation cohort (n = 124) at a 7:3 ratio. The method to filter the genes is the same as before, firstly, Univariate Cox regression analysis was performed to assess genes significantly associated with PFS (p < 0.05).

**Figure 10:**
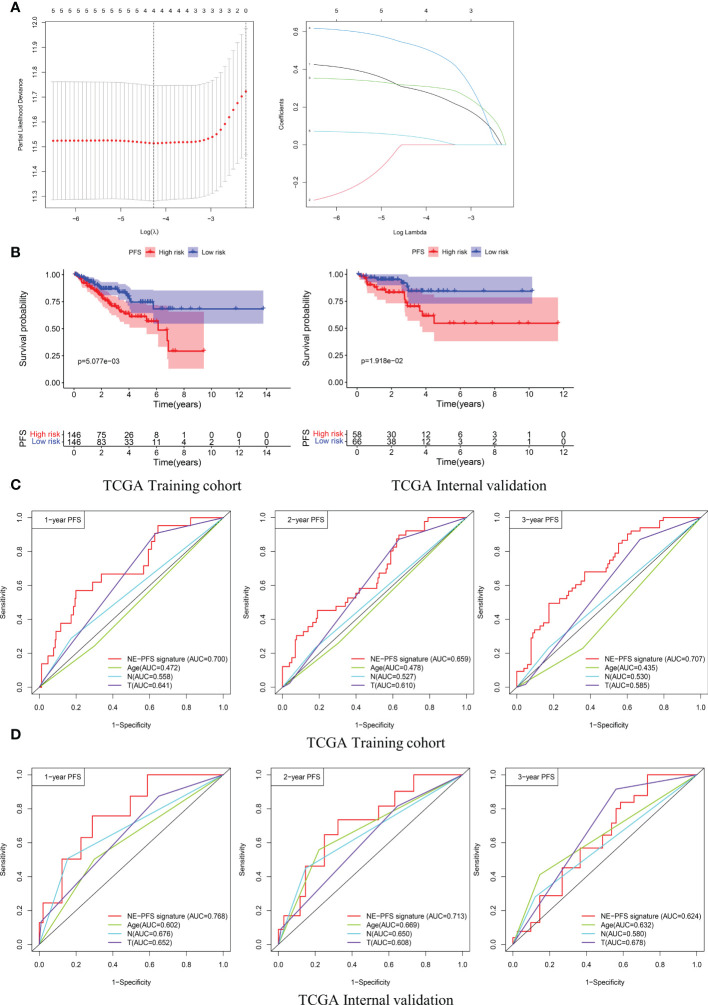
Construction and validation of the prognosis model for PFS in the TCGA PanCancer cohort. **(A)** Four genes correlated with PFS were selected for multivariate analysis by LASSO regression analysis. **(B)** Kaplan-Meier plots evaluate the predictive ability of the constructed prognostic model in the TCGA PanCancer training cohort and internal validation cohort, respectively. **(C, D)** NE-PFS signature exhibited better predictive ability than other clinical features as displayed, the 1-, 2- and 3- year AUC for PFS was 0.700 (95% CI: 0.587−0.814), 0.659 (95% CI: 0.566−0.752), and 0.707 (95% CI: 0.622−0.792) in the TCGA PanCancer training cohort.

Subsequently, the LASSO method by glmnet (version 4.0.2) R package for variable selection (**Figure 10A**). Ultimately, 3 genes, including *STMN1, UBE2S* and *HMGN2* were recognized as NE-PFS signature *via* multivariate Cox and Stepwise Algorithm.

NE-PFS signature score = expression level of 0.302 * *STMN1* + expression level of 0.391 * *UBE2S* + 0.653 * *HMGN2*. The process of building the model has been described in detail above. Compared with the low risk, Kaplan-Meier plots elucidated that the high risk had worse PFS (**Figure 10B**), p < 0.05). The AUC curve presented with decent result in predicting the PFS in training cohort (AUC for 1-, 2-, and 3 years PFS: 0.700 (95% CI: 0.587−0.814), 0.659 (95% CI: 0.566−0.752), and 0.707 (95% CI: 0.622−0.792)) (**Figure 10C**), then the predictive model was then validated in the internal TCGA PanCancer validation cohort (**Figure 10D**).

### Construction of nomograms

**Figure 11:**
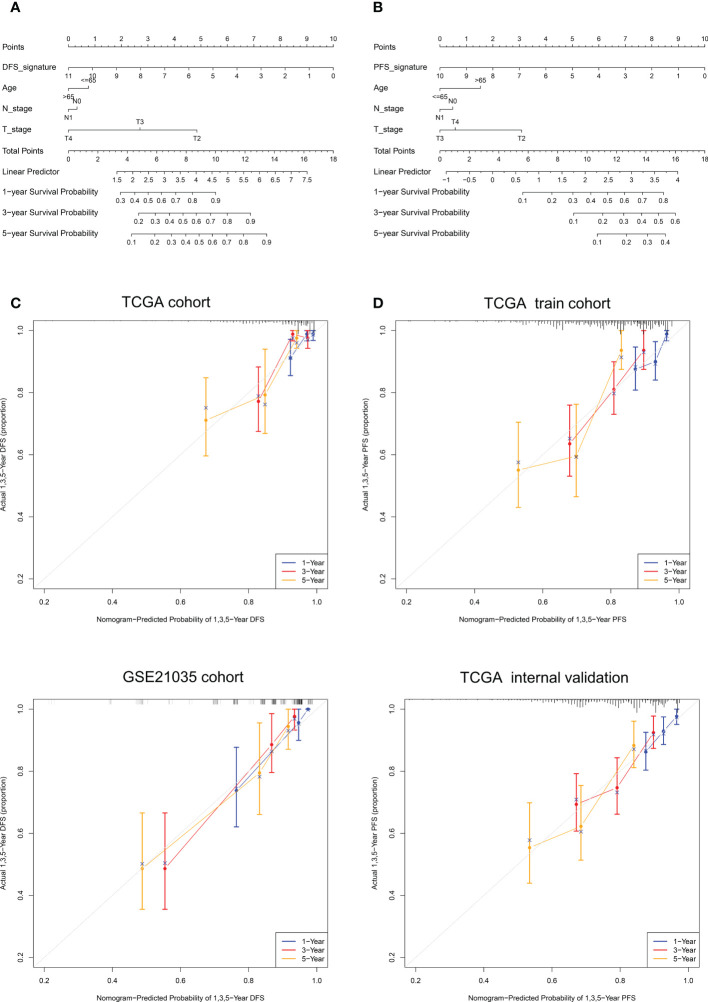
Nomogram construction and calibration plot validations for DFS and PFS prediction in PCa. **(A, B)** The composite nomogram consists of the DFS- or PFS- signature and clinical features of the individual patient, by adding the points from variables listed together, the 1-,3- and 5-year survival (DFS or PFS) probability can be inferred by the clinician. **(C, D)** Calibration curves for validation the consistence between 1-, 3- and 5-year (blue, red and orange color, respectively) inferred DFS and actual data in TCGA cohort and GSE21035 cohort. The dashed line represents the best match between the nomogram-predicted probability and the actual data evaluated by Kaplan–Meier analysis.

It can be concluded from the above analysis that the NE-DFS signature and NE-PFS signature could independent prognostic indicators for PCa patients. In addition, age, race, tumor stage, gleason scores were also incorporated in the nomogram tool to predict the outcome of individual patients (1, 3 and 5-year DFS and PFS probabilities of PCa in the TCGA PanCancer cohort (**Figure 11A**). Then, on basis of the total point (the sum score of each variable), the rate of DFS and PFS at 1-, 3- and 5-year can be inferred. In addition, the line-segment in the calibration plots was close to the 45°C line, the model’s predictions of 1-, 3- and 5-year DFS and PFS probabilities were favorably consistent with the ideal predictions (gray line) in both training cohort and validation cohort (**Figure 11B**), indicating that the nomogram model could be used as reliable indicator to predict DFS and PFS in CRC patients. In addition, we also mapped the calibration curves of the prognosis model. **Figure 11C** and **D** showed the calibration curves of recurrence-free survival model and progression-free survival model, respectively.

## Discussion

Secondary CRPC and even NEPC emerge as one of the most important killers threatening men’s health (50). In present study, Single-cell RNA seq and bulk RNA seq samples were used to discovered 12 differential genes characterized by CRPC-NE, the subsequent result demonstrated that a six−gene diagnostic signature (*HMGN2, MLLT11, SOX4, PCSK1N, RGS16* and *PTMA*) could serve as a reliable predictor to distinguish CRPC-NE from CRPC. Furthermore, we observed that there exists specific ligand–receptors among 16 cell types recognized, including ANGTP, CXCL, IGF, IL16, CSF, LIFR, OSM, and PROS pathways.

As is well-known, macrophage migration inhibitory factor (MIF) is involved in many carcinogenic processes, including cell proliferation, angiogenesis and inhibition of host tumor cell immune surveillance (51, 52). Experiments in LNCaP sublines indicated that during neuroendocrine differentiation, although MIF synthesis decreased, MIF release significantly increased, which may promote cancer progression or recurrence especially after androgen deprivation (53). It can be seen from **Figure 6C** that there exists strong intercellular communication between NEPC_luminal/NE cells and T cells, B cells, plasma cells and monocytes *via* MIF (Macrophage migration inhibitory factor) pathway, where the ligand receptor pairs involved are MIF-(CD74+CD44)and MIF-(CD74+CXCR4). It is worth mentioning that CXCR4 may form a functional MIF receptor complex with CD74, mediating MIF-stimulated, CD74-dependent AKT activation (54), In addition, *in vivo* and *in vitro* experiments showed that the inhibition of CXCR4 reduced the aggressiveness and chemosensitized PCa cells (55, 56), showing that MIF-(CD74+CXCR4)axis can be used as the target of comprehensive treatment.

Most importantly, immune cell infiltration and GSVA analysis showed that there were also significant differences between CRPC and NEPC in KEGG pathways and immune cell abundance. Drug metablism cytochrome p450 pathway attracts our attention greatly, Cytochrome P450 protein is a monooxygenase involved in the synthesis of cholesterol, steroids and other lipids (57). Drug resistance to ADT such as abiraterone may be caused by overexpression or mutation of *CYP17A1*, increased upstream substrate synthesis, or increased drug metabolism or efflux (58). Studies in LNCaP cells and xenografts have shown that the enzymes required for *de novo* steroidogenesis (including *CYP17A1*) are increased in castration resistance sublines and can produce detectable androgen levels (59–61). Consistently, our study shows that cytochrome P450 pathway is highly expressed in CRPC. In addition, Maayan and Antonio’ results showed that the production of dihydrotestosterone by neural-like cells was increased in mice in a *CYP17A1* independent manner under castration conditions (62, 63), accounting for the low expression of cytochrome P450 pathway in CRPC-NE to some extent (**Figure 5E**). Indeed, there is increasing evidence that prostate cancer cells transdifferentiate into neuroendocrine phenotypes and appear to be strongly induced in an androgen depleted environment (26, 64–66).

In our study, *HMGN2, MLLT11, SOX4, PCSK1N, RGS16* and *PTMA* were newly explored to predict the characteristics of CRPC-NE. Zhang et al. focused only on the bulk-RNA level, which may ignore the differences within the samples. Secondly, the samples with insufficient information are not filtered, resulting in bias consequently, our research overcomes these shortcomings. Previously, as an important developmental transcription factor, sex-determining region Y-box 4 (*SOX4*) proved to be combined with promoters to regulate genes closely related to neuroendocrine prostate cancer, including canonical *EZH2* (67, 68). Our research and previous studies have shown that the expression level of SOX4 increased with the progress of PCa, significantly higher in NEPC compared with CRPC (**Figures 5B**, **7B**) (26, 69, 70). Current experiments also verified that SOX4 knockdown could reduce the proliferation of LNCaP-NEPC cells and inhibit the expression of NEPC markers (71). Prothymosin alpha (PTMA/ProTα) is widely expressed in many tissues and highly conserved in mammalian RNA sequences (**Figure 8C**) (72). Suzuki et al. demonstrated that the expression level of *PTMA* increased with the progression of normal epithelium, prostatic intraepithelial neoplasia (PIN) to prostate cancer, and was positively correlated with Gleason grade and clinical stage (73), but the relationship with NEPC was unknown.

When it comes to *HMGN2, MLLT11, PCSK1N* and *RGS16*, the diagnostic performance of them for NEPC has not been shown, deacetylation of high mobility group nucleosomal binding domain 2 (*HMGN2*) enhances STAT5A transcriptional activity, thereby regulating prolactin induced gene transcription and breast cancer growth (74, 75). Additionally, AZD1480 inhibits the growth of recurrent castration resistant CWR22Pc xenograft tumors by targeting JAK2-STAT5A/B signal transduction was observed in another study (76). Consequently, it is worth exploring the relationship between *HMGN2* and JAK2-STAT5A/B pathway. Involvement of *MLLT11* promoted the progression of ovarian cancer, bladder cancer and endometrial cancer in previous study (77, 78). Moreover, the granule protein family member *PCSK1N*, also known as ProSAAS, is a protein produced almost entirely by a wide variety of endocrine, neuronal and neuroendocrine cells (79, 80). Recently, the proteolytic neuropeptide PEN derived from the precursor ProSAAS has been identified as a selective, high affinity endogenous ligand for the orphan receptor GPR83. Both of them show regional specific expression in neuroendocrine tissues and may be used as a target for the treatment of neurological and immune diseases (81). Moreover, it is well acknowledged that the abnormal activity of phosphatidylinositol 3-kinase (PI3K) pathway supports the growth of many tumors, including breast, lung and prostate tumors. Studies have shown that G protein signaling 16 (*RGS16*) can act as a tumor suppressor by inhibiting the growth of PI3K dependent breast epithelial cells (82), while inhibiting PI3K/AKT downregulates REST expression and induces NE markers in LNCaP, PC3 and LNCaP95 cells (83). It is known that NEPC has great heterogeneity, integrating these different datasets to deduce 6 markers to predict the characteristics of CRPC-NE may be debatable. Actually, in order to reduce errors, we have eliminated atypical neuroendocrine prostate cancer including Paneth cell neuroendocrine differentiation, large cell neuroendocrine carcinoma, carcinoid, mixed samples and so on to reduce the heterogeneity within NEPC samples in order to produce more reliable biomarkers. What’s more, because of the limited sample size in the public database, we are also collecting corresponding data in clinical work. We plan to carry out Bulk-RNA sequencing and SC-RNA sequencing on the same batch of CRPC and NEPC samples, and deduce biomarkers from the SC-RNA and Bulk-RNA sequencing data of the same batch of samples and verify them, so as to better reveal the similarities and differences between CRPC and NEPC.

Regarding the NE-DFS signature and NE-PFS signature, the former can accurately predict DFS in PCa patients, and shows significant survival differences between low-risk group and high-risk group. It also shows excellent AUC values in GSE20135 (n=138) validation set, with AUC values of 0.899 (95% CI: 0.806−0.992), 0.843 (95% CI: 0.746−0.941) and 0.810 (95% CI: 0.712−0.907) for 1-, 2-, and 3-year DFS, respectively, which is significantly higher than the predictive ability of Gleason score and tumor stage. Previous researchers have used multivariable Cox regression analysis to obtain 22 autophagy related genes and build DFS prognosis model, although the AUC value of the prognosis model reached 0.85, there were too many biomarkers, which greatly reduced the clinical practicability (84). On the contrary, although our model only contained two genes (*STMN1* and *PCSK1N*), it still had high accuracy for clinical application. In Wang study, we can observe that the 1- and 3-year prognostic accuracy of AUC is 0.765 and 0.698 in the training cohort, 0.715 and 0.713 in the validation set, respectively (85). As for the NE-PFS signature composed of three markers (*STMN1, UBE2S* and *HMGN2*), the results showed that there was a significant difference in the survival rate between the low- and high-risk groups in the training cohort (p = 0.005077) and internal validation cohort (p = 0.01918), and the AUC curve of the prediction model at 1-, 2-, 3-year was greater than 0.65. However, due to the limited number of our samples, additional samples are needed to verify the robustness of the above model. We also actively recruit qualified patients and plan to make further verification. Secondly, the molecular mechanism of how the NE-DFS signature and NE-PFS signature affect the prognosis of PCa needs to be clarified through further clinical research.

## Conclusion

In the present study, A robust signature composed of six genes for screening CRPC-NE were developed. In addition, we constructed and verified the DFS and PFS prognostic model for prostate cancer patients and the KEGG pathway difference as well as tight intercellular communication between CRPC and CRPC-NE were also further discussed, which is helpful to better guide clinical work.

## Data availability statement

The datasets presented in this study can be found in online repositories. The names of the repository/repositories and accession number(s) can be found in the article/supplementary material.

## Ethics statement

Ethical review and approval was not required for the study on human participants in accordance with the local legislation and institutional requirements. Written informed consent for participation was not required for this study in accordance with the national legislation and the institutional requirements.

## Author contributions

JL and ZZ conceived and designed the study. JL and YC were responsible for data collection, collation and statistical analysis with bioinformatics methods. ZW, YM and JP carried out data interpretation and chart drawing. JL and YC wrote the manuscript, which was further polished and confirmed by YL and ZZ. All authors contributed to the article and approved the submitted version.
